# Supplementary figures and images for: The FASTK family proteins fine-tune mitochondrial RNA processing
Source: PLoS Genet. 2021 Nov 8;17(11):e1009873. doi: 10.1371/journal.pgen.1009873 (PMC8601606; doi:10.1371/journal.pgen.1009873)

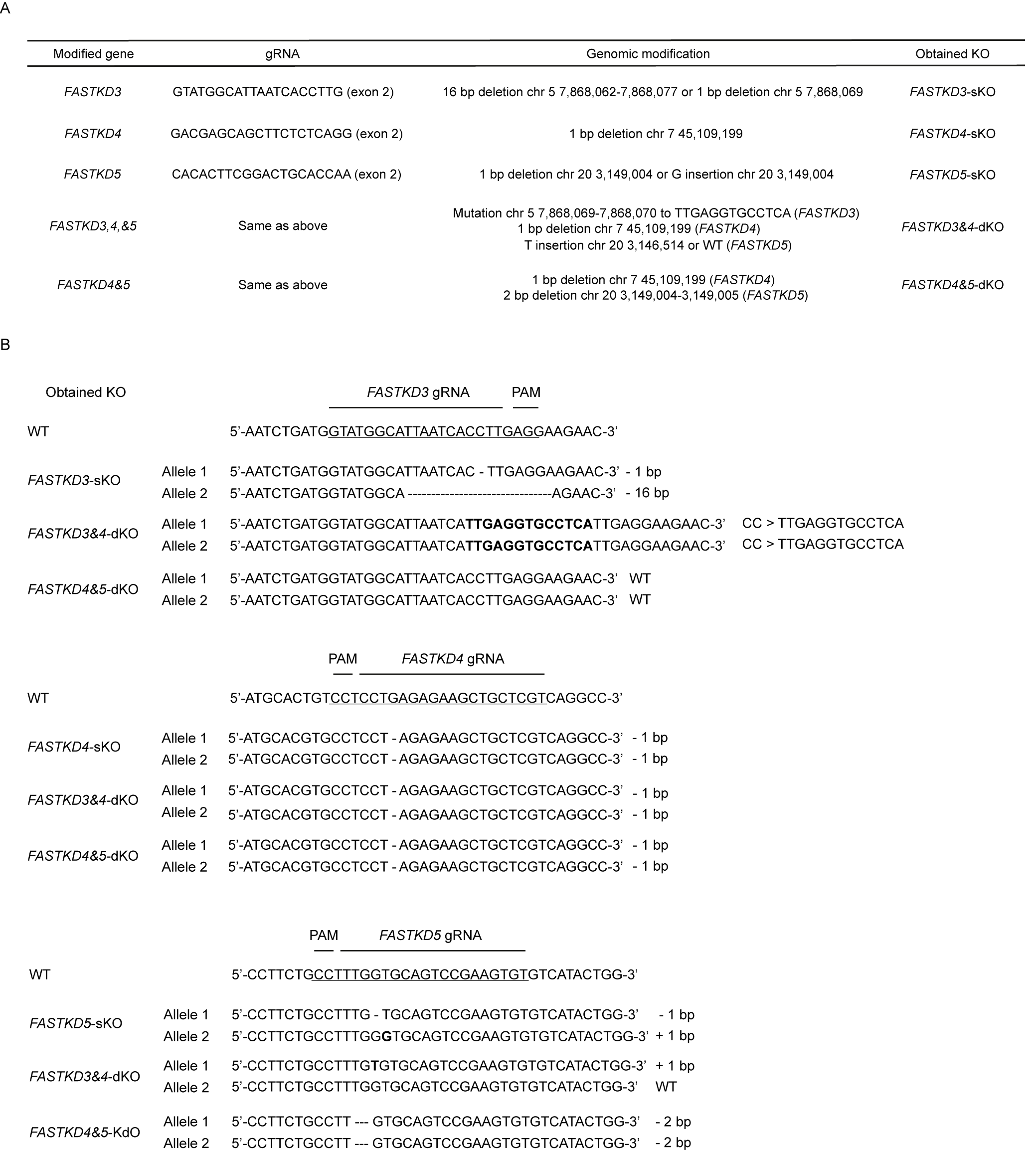

Supplement: S1 Fig — Genomic modifications shown in (A) were detailed in (B). (TIF) [file pgen.1009873.s001.tif]

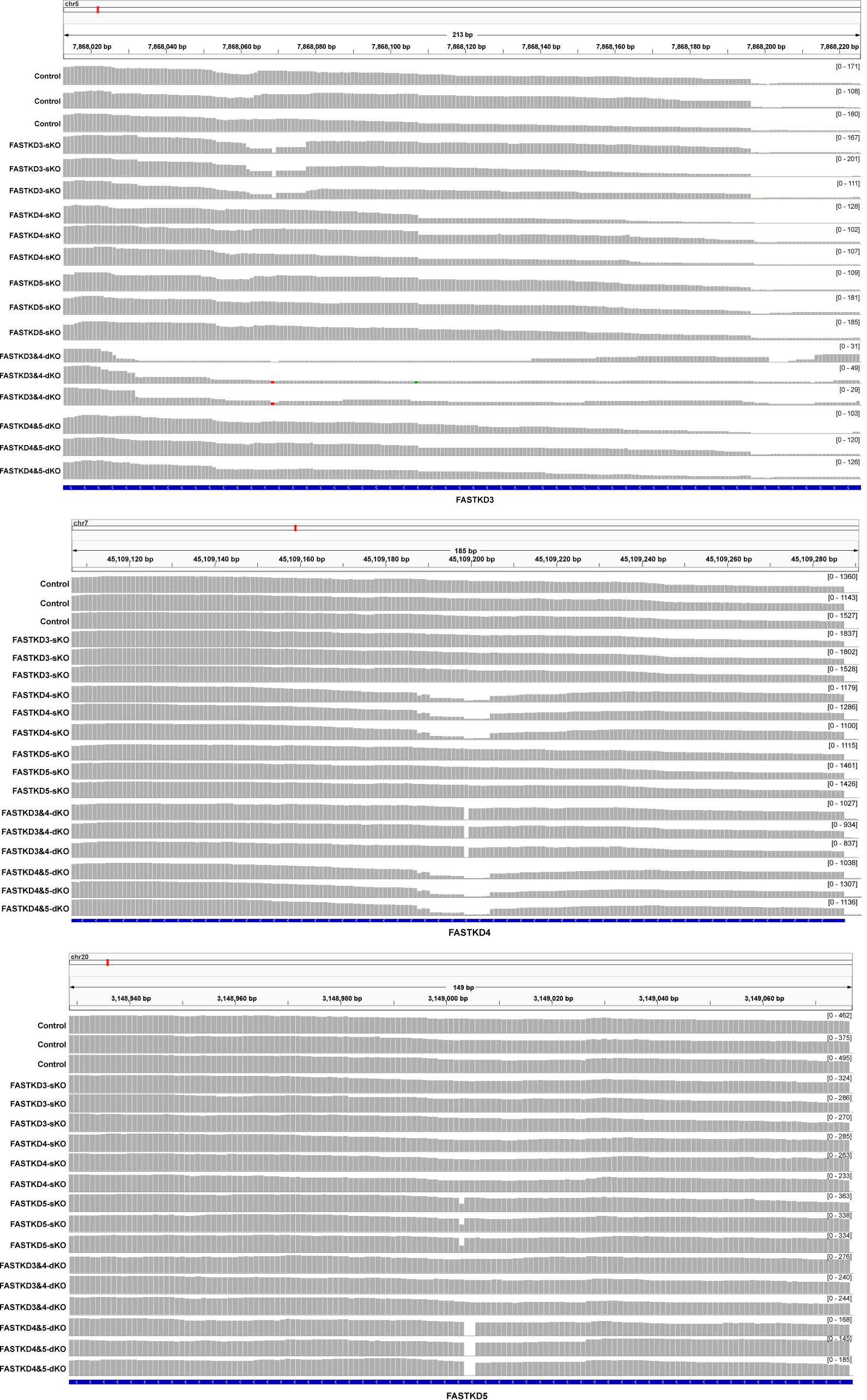

Supplement: S2 Fig — Specific deletions in each of the targeted FASTKD genes in each of the HAP1 cell lines relative to the control HAP1 cell line were shown. (TIF) [file pgen.1009873.s002.tif]

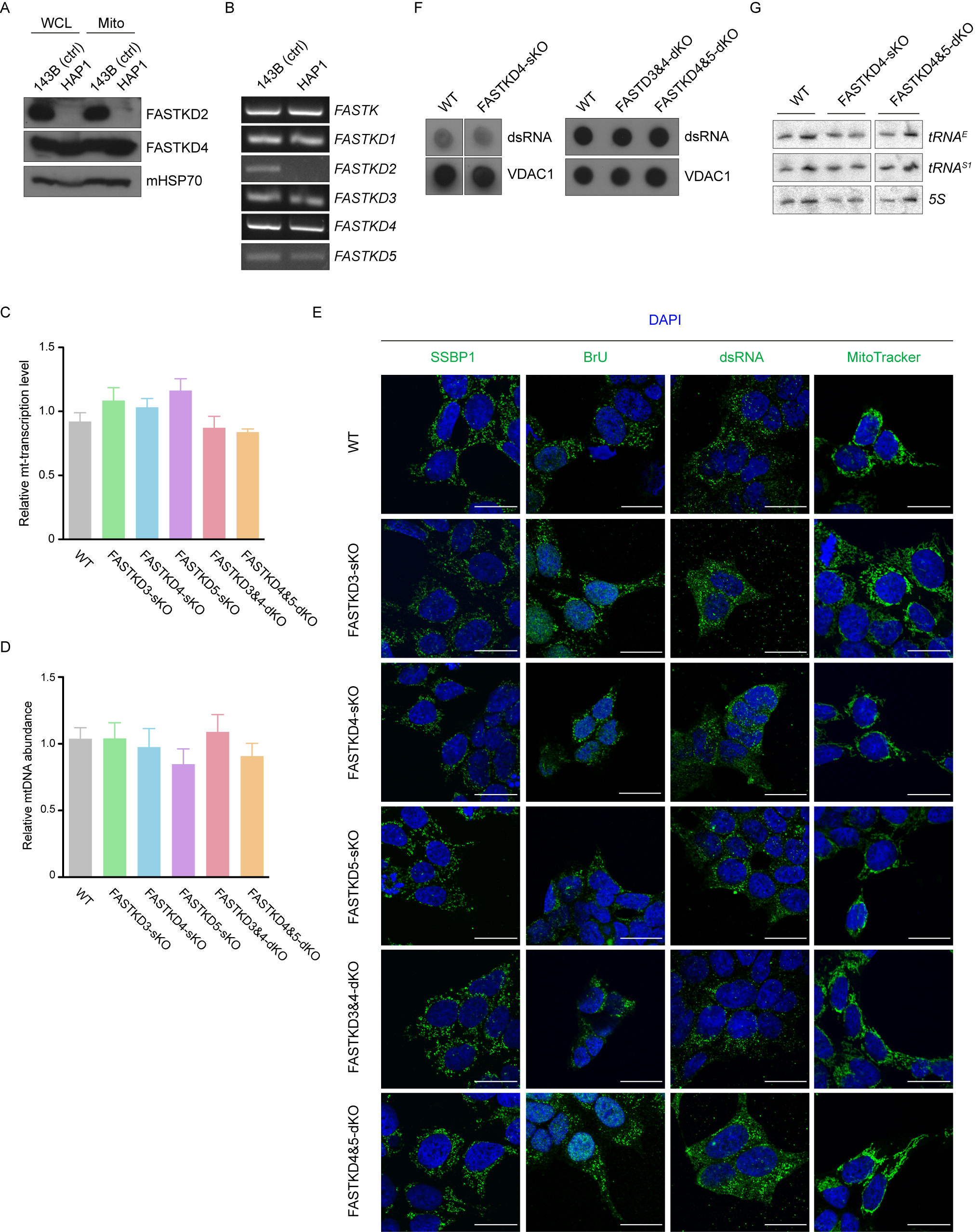

Supplement: S3 Fig — (A) Protein expression levels in 143B (control) and WT HAP1 cells were assessed by immunoblotting. Whole cell lysates (WCL) and mitochondria extracted from the cells were analyzed. mtHSP70 was used as a loading control. (B) mRNA expression levels in 143B (control) and WT HAP1 cells were analyzed by RT-PCR. (C) Relative abundance of 7S RNA in WT (control) and FASTKDs-KO HAP1 cells were quantified by RT-qPCR (n = 4). The amounts of 7S RNA were normalized by GAPDH. (D) The relative abundance of mtDNA in the FASTKDs-KO cells compared to controls was measured by qPCR and normalized to total nuclear DNA (n = 3). (E) WT and FASTKDs-KO HAP1 cells were labeled with BrU, MitoTracker Red, and DAPI, and immunolabeled with anti-SSBP1, anti-BrU and J2 anti-dsRNA. Immunofluorensce was analyzed by confocal microscopy. Scale bars indicate 20 μm. (F) The amount of mitochondrial dsRNA in the FASTKDs-KO cells and controls was measured by dot blotting. VDAC1 protein was used as a loading control. (G) Steady-state levels of mitochondrial tRNAs in WT (control) and FASTKDs-KO HAP1 cells were measured by northern blotting. 5S was analyzed as a loading control. (TIF) [file pgen.1009873.s003.tif]

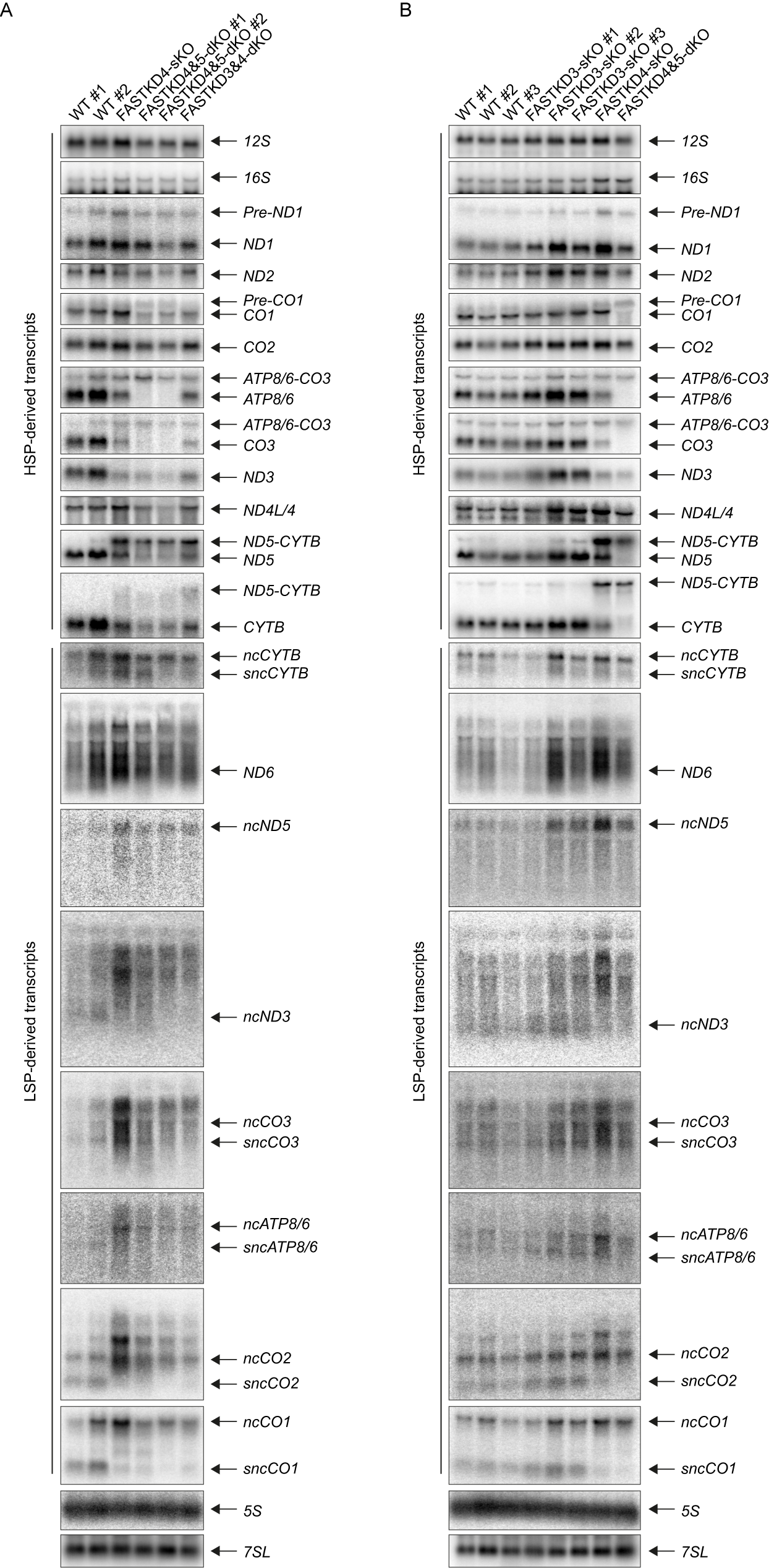

Supplement: S4 Fig — Related to Fig 2. Two entire gels/exposures are shown. 5S and 7SL were used as loading controls. Equal amounts of total cellular RNA were loaded on the gel. Independent biological samples were analyzed per genotype. The pair of WT #2 and FASTKD4-sKO (A), a pair of WT #2 and FASTKD3&4-dKO (A), a pair of WT #1 and FASTKD4&5-dKO (B), and a pair of WT #3 and FASTKD3-sKO (B) are also shown in Fig 2A. (TIF) [file pgen.1009873.s004.tif]

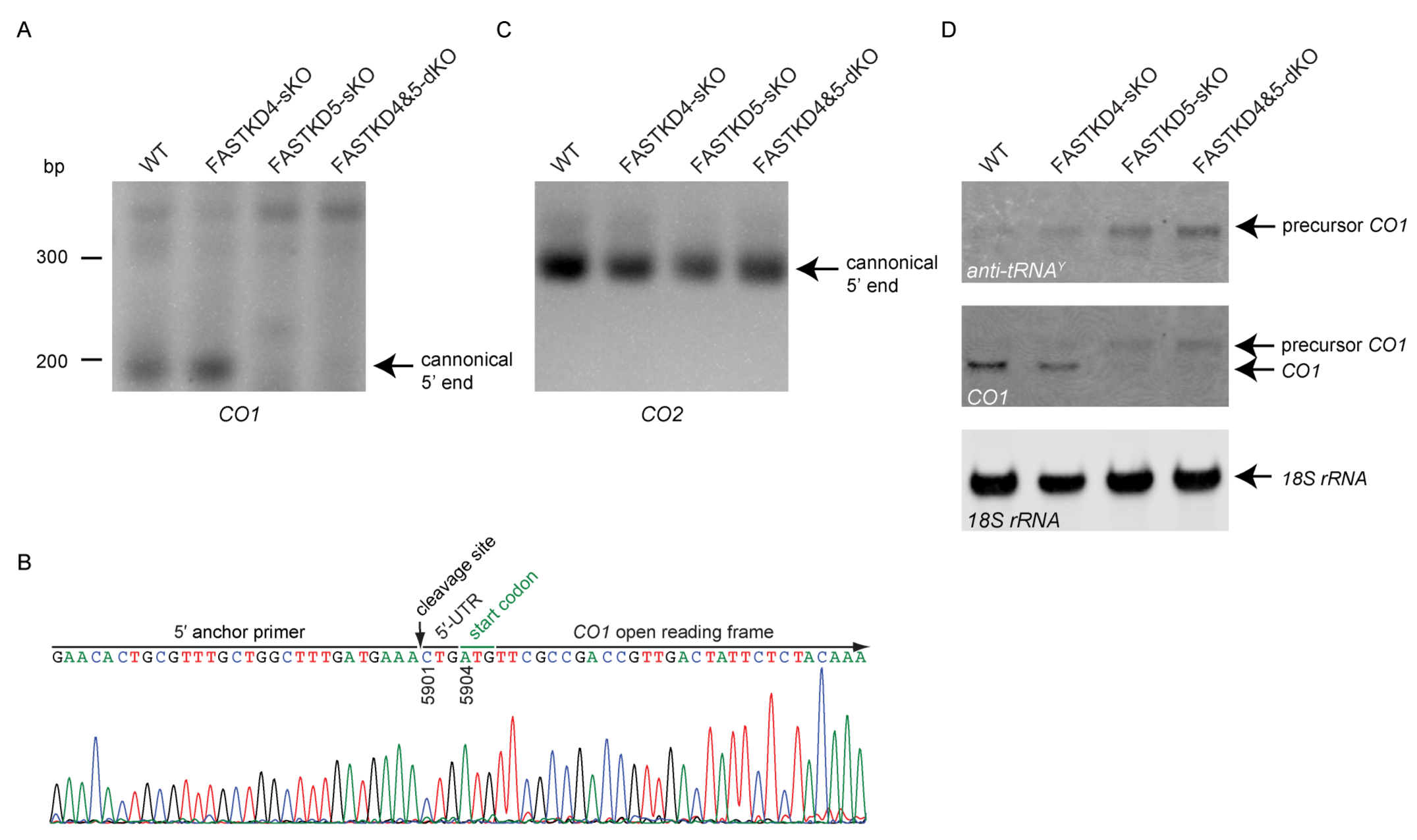

Supplement: S5 Fig — (A) RACE of the 5′ UTR of CO1 mRNA in WT (control) and FASTKDs-KO HAP1 cells. (B) Sanger sequencing of the 5′ UTR of CO1 mRNA from WT HAP1 cells. (C) RACE of the 5′ end of CO2 mRNA in WT (control) and FASTKDs-KO HAP1 cells. (D) Northern blotting of RNA isolated from WT (control) and FASTKDs-KO HAP1 cells against the antisense tRNAY, CO1 mRNA, and 18S rRNA that was used as a loading control. (TIFF) [file pgen.1009873.s005.tiff]

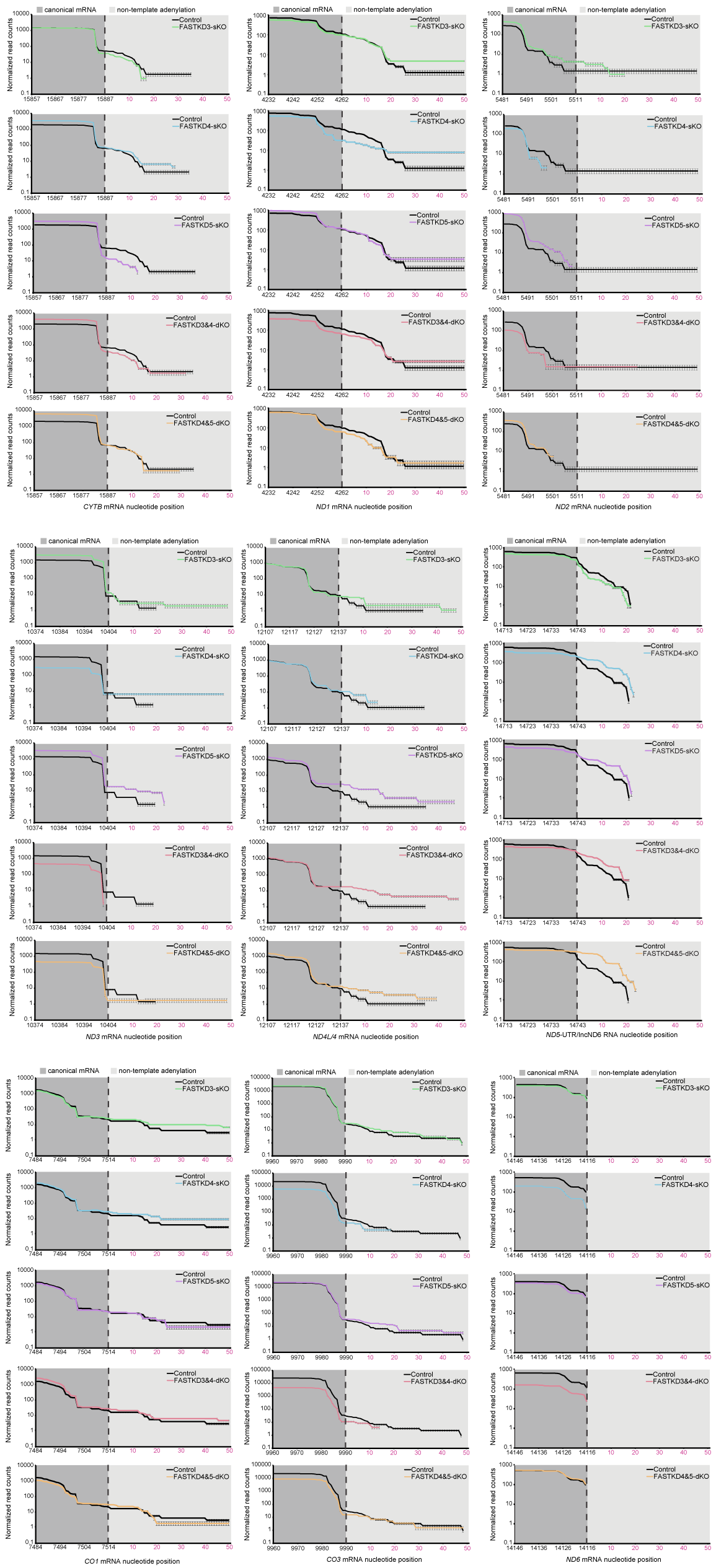

Supplement: S6 Fig — Related to Fig 4. Mapping of RNA-seq reads to polyadenylated mitochondrial mRNA reference sequences was used to analyze the polyadenylation status of mRNAs in the FASTKDs-KO cell lines compared to controls. The canonical 3′ region of each mRNA is shown in dark grey and the section of polyadenosine is shown in light grey. (TIF) [file pgen.1009873.s006.tif]

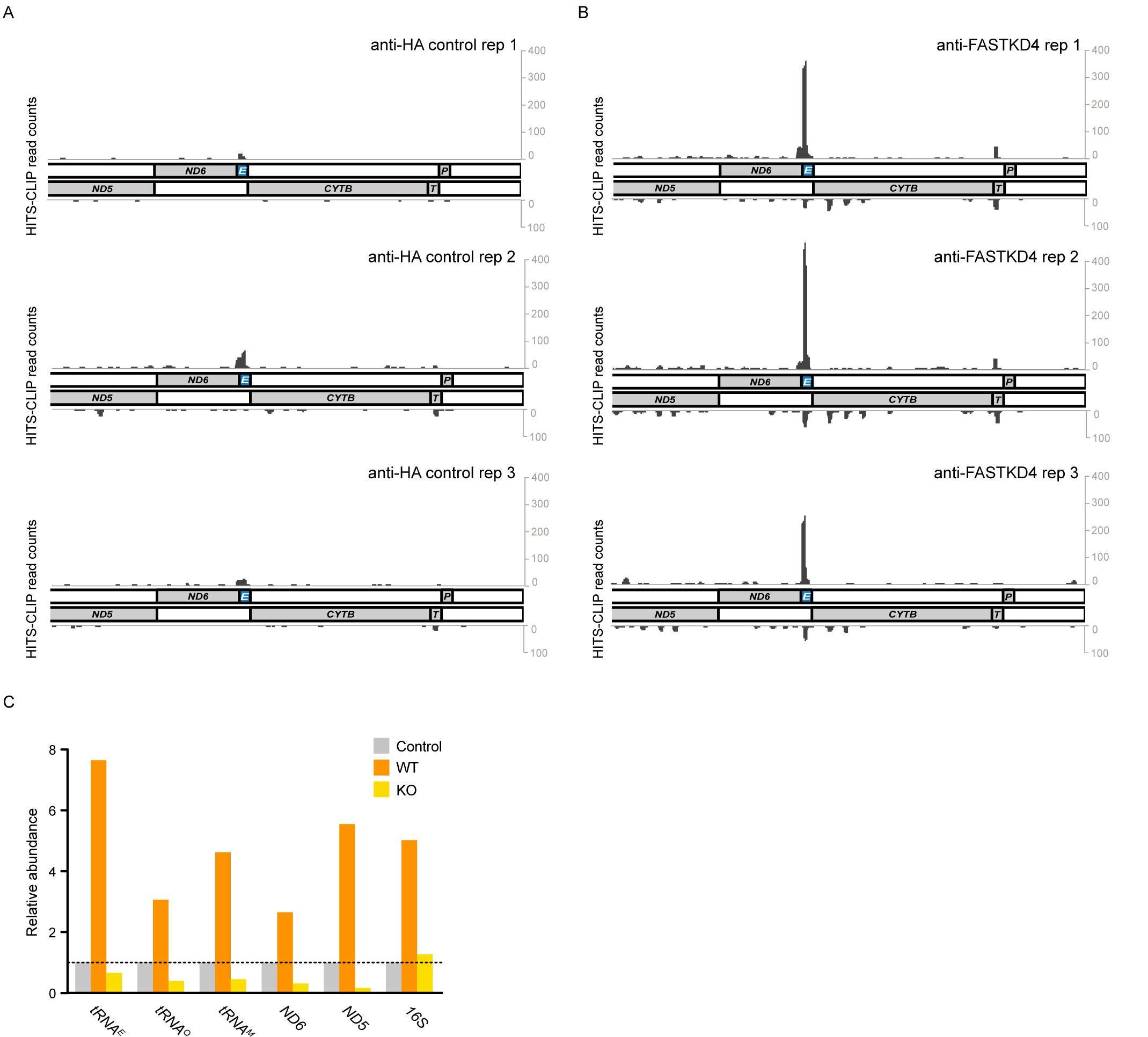

Supplement: S7 Fig — Related to Fig 5. (A-B) Strand specific HITS-CLIP read counts corresponding to the 13401–16569 region of the mtDNA for control (anti-HA antibody) (A) and anti-FASTKD4 antibody (B). (C) The tRNAE enriched through FASTKD4 immunoprecipitation from WT and FASTKD4-sKO HAP1 cells was quantified by strand-specific RT-qPCR. Anti-Flag antibody was used a s control. A representative result from two independent biological experiments is shown. (TIF) [file pgen.1009873.s007.tif]

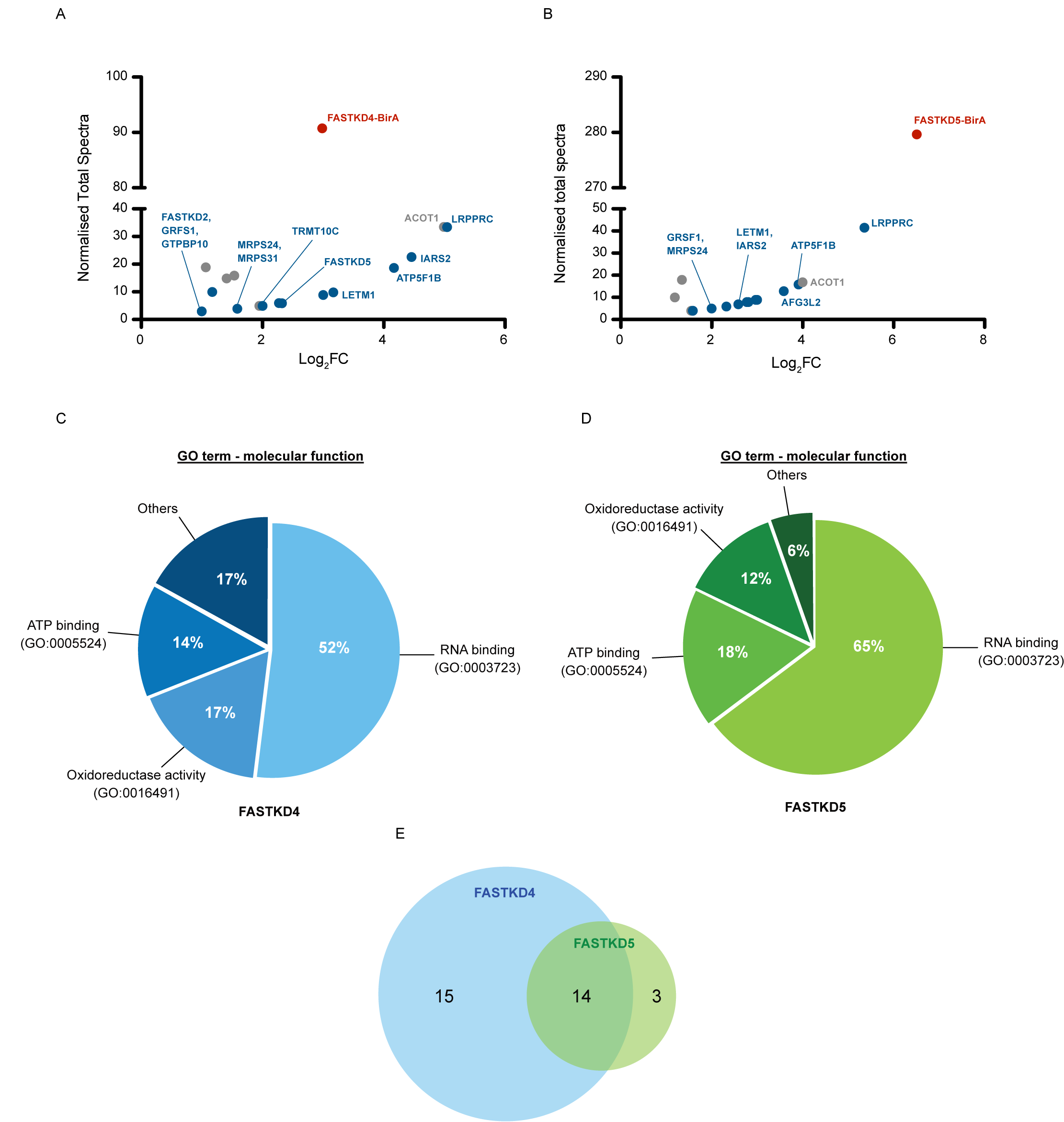

Supplement: S8 Fig — Log2FC (FASTKD4-BirA/control) (A) or Log2FC (FASTKD5-BirA/control) (B) was plotted against a sum of normalized total spectra. Only significantly enriched proteins are shown (Log2FC ≥ 1). Cells that do not express the bait protein were used as control. Mitochondrial proteins are shown in blue. Red dot represents BirA-tagged bait proteins. (C-D) Gene ontology analysis (GO—molecular function) of mitochondrial proteins identified by the BioID of FASTKD4 (C) and FASTKD5 (D). (E) Number of mitochondrial proteins found in either or both BioID data sets from FASTKD4 or FASTKD5. (TIF) [file pgen.1009873.s008.tif]

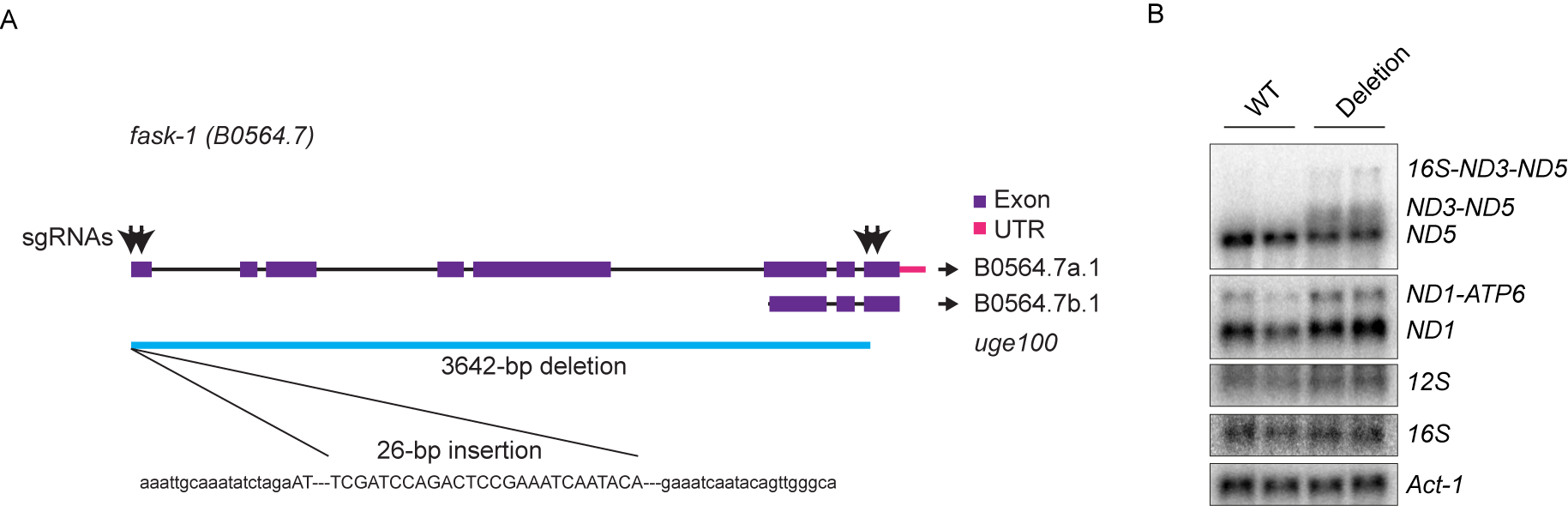

Supplement: S9 Fig — (A) A schematic representation of fask-1 (B0564.7) deletion allele generated using CRISPR/Cas9 technology. Two sgRNAs each were used to cleave the genomic DNA near the start codon and in the last exon of the FASK-1 locus. The deletion allele fask-1(uge100) contains a truncated start codon, a 26 bp insertion and a 3642 bp deletion that removes all of the FASK-1 coding sequence except 153 bp of the last exon. (B) The steady-state levels of mitochondrial transcripts in WT (control) and FASK-1 deletion strains were measured by northern blotting. Act-1 was used as a loading control. (TIF) [file pgen.1009873.s009.tif]

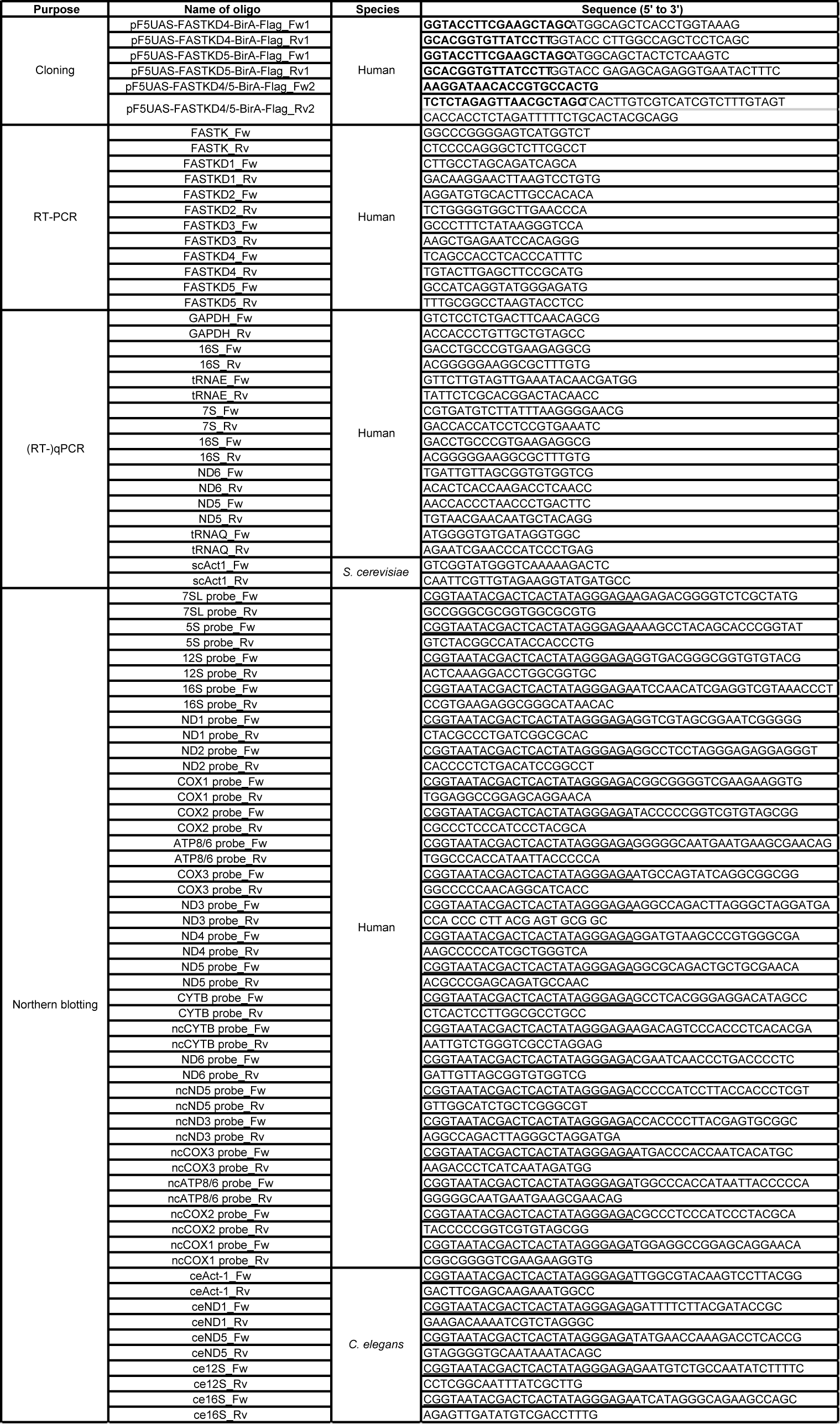

Supplement: S1 Table — Sequences used for Gibson assembly are shown in bold. T7 promoter sequence is underlined. (TIF) [file pgen.1009873.s010.tif]
