# Supplementary material for: The FASTK family proteins fine-tune mitochondrial RNA processing
Source: PLoS Genet. 2021 Nov 8;17(11):e1009873. doi: 10.1371/journal.pgen.1009873 (PMC8601606; doi:10.1371/journal.pgen.1009873)
Supplement: Attachment — Submitted filename: The manuscript describes the results of establishing cell lines in which the expression of one or two of the mitochondrial FASKD proteins is knocked down.docx [file pgen.1009873.s011.docx]

The manuscript describes the results of establishing cell lines in which the expression of one or two of the mitochondrial FASTKD proteins is knocked down. These proteins are involved in the RNA processing and post-transcriptional regulation of gene expression in the mitochondria. The work in this manuscript attempted looking and unraveling the function of each one of these family members in a comprehensive approach. The question of the control of gene expression of mitochondrial encoded genes is an important one and under an extensive study. Therefore, this manuscript would interest those who study mitochondrial gene expression, as well as those interested in RNA processing and stability. Although a defined clear picture of the mechanistic role of each member is still not at hand, this work is an important step towards this goal.

Major comments:

The authors use the terms “canonical” versus “non-canonical” transcripts throughout the manuscript. However, it is not clearly defined what is each one. Is a canonical transcript is the mature processed one or a transcript that is composed of the amino-acids coding region (and a polyA tail). For example, the matured processed CO1 has been mapped to have the tRNA^S^ antisense serving as its 3’ UTR. Similarly, the mature ND6 transcript has been mapped to have a long 3’ UTR of about 450 nt (Slomovic et al MCB 2005). Therefore, all the mitochondrial transcripts should be precisely defined and described as to their 5’ and 3’ ends, even if they are surrounded by tRNAs. This is also applied for the polyadenylation sites (see below).

For example, the term pre-CO1 is used but no description of what exactly is that RNA molecule. The presented RACE results are of low resolution gel and the nt sequences of the RACE clones are not shown.

The same account to Fig 7 where the RNA molecules are schematically presented but the reader cannot identify the exact transcripts. These should be indicated using the mit genomic nt sequence numbering or a similar mapping system.

Fig 4 and S5 Fig. It is not clear what is presented here. Is the canonical mRNA region ends at the translational stop codon or at the mature 3’ end of the transcript? As said, these are not the same for several transcripts. Is the section of polyadenylation representing a polyA sequence or the 3’ UTR of the RNA / the seq 3’ to the CDS? This is not explained in the legends to figures and the ref indicated does not seems to be the right one. In addition, the title of Fig 4 seems to be not the right one and in the legend to S5 Fig it is indicated “reveals effects on polyadenylation in all mRNAs” which is not the case. For example, the last column in S5 Fig present the ND6 mRNA polyadenylation. This mature transcript is about 950 nt long and the polyadenylation is at the 3’ end. Only nt 445 to 525 sequence is presented here so where is the mature 3’ end?

Minor:

1. S3E Fig. The Bru and dsRNA staining is very faint and hard to detect.
2. What is the ND5-cytB junction? Define it clearly.
3. S4C Fig. The blot of the pre-CO1 is too faint.
4. Ref 64 does not seem the right one for the polyadenylation method.
