# Supplementary material for: The FASTK family proteins fine-tune mitochondrial RNA processing
Source: PLoS Genet. 2021 Nov 8;17(11):e1009873. doi: 10.1371/journal.pgen.1009873 (PMC8601606; doi:10.1371/journal.pgen.1009873)
Supplement: Attachment — Submitted filename: Response to reviewers.docx [file pgen.1009873.s012.docx]

We would like to thank the referees for their insightful comments and suggestions on how to improve our manuscript. We think that we have answered their concerns and that our manuscript has been significantly strengthened as a result.

**Response to the editors**

1) “Given the haploid state of the cell line used, I would appreciate if you could clarify why the identified *FASTKD5* variant in the *FASTKD3&4*-dKO cells is deemed as irrelevant to the experiments. The western blot in Figure 1 would suggest otherwise.”

We selected the HAP1 cell line because of its nearly haploid karyotype, and because all of the *FASTK* family genes are located within the haploid regions of the HAP1 cells. We unexpectedly found that the cell line had spontaneously become diploid in chromosomes containing the *FASTKD3* and *FASTKD5* genes (S1 Fig). According to Horizon Discovery, the HAP1 cell line is genetically unstable and spontaneously becomes diploid over time (see <https://horizondiscovery.com/en/frequently-asked-questions/cell-lines/are-hap1-cell-lines-genetically-stable-ie-do-they-stay-haploid>).

The *FASTKD3&4*-dKO cell line carried a mutation in one of the *FASTKD5* alleles (S1 Fig). However, this did not change the expression of FASTKD5 protein (Fig 1B and Fig I below), therefore this cell line was used as a *FASTKD3&4*-dKO. Furthermore, this *FASTKD5* mutation didn’t show any phenotype (Fig 2-4, and 6), including its previously established role in non-canonical RNA processing in Antonicka *et al* Cell Reports 2015 (Fig 2 and 3).

Fig I. Expression levels of FASTK family proteins and mtRBPs involved in the post-transcriptional regulation in WT (control) and *FASTKDs*-KO HAP1 cells were measured by immunoblotting. γ-tubulin was used as a loading control for whole cell lysates. mtHSP70 and VDAC1 were used as mitochondrial loading controls. These data are independent biological replicates of the results shown in Fig 1B.

2) “The conclusions of the Northern blot experiments in Figure 2 seem to be based on statistical significance, rather than physiological relevance. Some of the signals are very low, making quantification difficult, increasing variation and therefore potentially skewing the conclusions. The lack of error bars for WT samples in Figures 2B-E, makes interpretation more difficult. This is especially true for some of the nc and sncRNA species. It woudl be helpful in some cases, such as for *ND6* or *ncND3*, to indicate what parts of the blot were used for quantification. Please ensure the readers in the figure legend that the presented gel sections in Figure 2A stem from the same representative gel and not from different gels. It might also be useful to provide one set of images in the supplementary files.”

We improved the representation of Fig 2: 1) all the signals used for quantification are clearly marked with arrows, 2) cut blots cover wider areas, and 3) the experimental set up is described in greater detail in the figure legend.

Due to technical limitations, it would be inaccurate to show error bars for WT samples. Since we had a large number of samples to handle, we had to load biological replicates on different gels in some cases. Therefore, the signal intensities that we quantified using the Typhoon imaging system (GE Healthcare) were done on samples labelled on the same day and resolved on the same gels. We calculated the changes within individual gels and we now provide additional biological replicates of our results in Figure II (see below) to show that the results are consistent between independent biological experiments.

Fig II. The steady-state levels of rRNAs, mRNAs and ncRNAs in WT (control) and *FASTKD5*-KO HAP1 cells were measured by northern blotting. *5S* and *7SL* were used as loading controls. Equal amounts of total cellular RNA were loaded on the gel. Independent biological samples were analyzed in triplicates per genotype.

3) “I recommend checking the manuscript for inconsistencies. For instance, FASTKD5 levels in *FASTKD3&4*-dKO (line 140) and *FASTKD3*-sKO cells (line 157). Interaction between FASTKD3 and FASTKD5 (line 159), but not in BioID data It would help the reader to clarify that the BioID was performed in a polyploid cell line, which expresses FASTKD2, unlike the haploid cell line used throughout the manuscript. The reader might otherwise wonder how an interaction with in the FASTKD4-BirA BioID experiment was possible (Fig. S7).”

We agree with these suggestions and have made clarifications in the revised manuscript.

Based on the instability of FASTKD5 in *FASTKD3*-sKO HAP1 cells and vice versa (Fig 1B and Fig I above), we raised the possibility that FASTKD5 and FASTKD3 proteins could interact each other. Indeed, it is often the case that when a protein from a complex is lost, other partners of the complex are destabilized and degraded. However, unlike in FASTKD3-sKO cells, FASTKD5 protein expression did not change in *FASTKD3&4*-dKO cells (Fig 1B and Fig I below), implying that FASTKD5 protein expression was upregulated to compensate the loss of FASTKD4 protein in the dKO cells. Nonetheless, we did not observe a physical interaction between FASTKD3 and FASTKD5 in the BioID analysis (S7 Fig). This inconsistency may be cell-type specific effects since 143B cells, which we used in the BioID analysis, express FASTKD2 protein unlike HAP1 cells (S3A Fig), and the expression of FASTKD2 may dynamically reprogram protein interaction profiles of the FASTK protein family in these cells. Additional experiments, including immunoprecipitation, should be performed in multiple cell lines to further test this hypothesis.

4) “Clarification of the RNAseq data and polyadenylation experiment will benefit clarity of the results.”

We have explained that we identified changes in the polyadenylation status of mitochondrial mRNAs using the RNAseq reads by mapping them to polyadenylated mRNA reference sequences. The RNAseq data were used to quantify the changes in mRNA reads and provide insight into the stability of each mRNA in response to specific *FASTKD* gene knockouts. Furthermore, we have already included an explanation on the use of RNAseq data to analyze effects on RNA processing. On page 8 we discuss that “The RNA-seq library construction approach enabled us to exclude mature tRNAs and retain unprocessed transcripts containing tRNAs that intersperse adjacent coding regions in the mitochondrial transcriptome.”

Further clarification of these plots is provided in response to Reviewer 2’s point 5.

5) “In general, the manuscript could benefit from clarity and less speculation in the results section.”

We have condensed the manuscript and removed unnecessary speculations in the results section.

6) It would help the reader if the canonical and non-canonical transcripts are defined and the transcript boarders described.

Please see the response to Reviewer 1’s comment 1)

**Response to reviewer #1**

1) “The authors use the terms “canonical” versus “non-canonical” transcripts throughout the manuscript. However, it is not clearly defined what is each one. Is a canonical transcript is the mature processed one or a transcript that is composed of the amino-acids coding region (and a polyA tail). For example, the matured processed *CO1* has been mapped to have the *tRNA^S^* antisense serving as its 3’ UTR. Similarly, the mature *ND6* transcript has been mapped to have a long 3’ UTR of about 450 nt (Slomovic *et al* MCB 2005). Therefore, all the mitochondrial transcripts should be precisely defined and described as to their 5’ and 3’ ends, even if they are surrounded by tRNAs. This is also applied for the polyadenylation sites (see below). For example, the term *pre-CO1* is used but no description of what exactly is that RNA molecule. The presented RACE results are of low resolution gel and the nt sequences of the RACE clones are not shown. The same account to Fig 7 where the RNA molecules are schematically presented but the reader cannot identify the exact transcripts. These should be indicated using the mit genomic nt sequence numbering or a similar mapping system.”

The exact ends of mitochondrial mRNAs are described in Temperley *et al.* Biochim Biophys Acta 2010, therefore we did not attempt to duplicate this work. Nevertheless, we have provided a chromatogram that precisely maps the cleavage site of *MT-CO1*, as we had done this previously for the *MT-ND6* transcript in Mercer *et al.* 2011, because this is an mRNA site of particular interest in our study.

We use canonical to mean those transcripts that are processed according to the tRNA punctuation model of mitochondrial RNA cleavage. On the other hand, we use non-canonical to mean those transcripts, *ATP8/6*, *CO1*, *CO3*, *CYTB*, *ND5* and *ND6*, all of which lack flanking tRNAs. Therefore, processing sites that are devoid of tRNA junctions are referred to as non-canonical processing junctions as clarified on page 3.

2) “Fig 4 and S5 Fig. It is not clear what is presented here. Is the canonical mRNA region ends at the translational stop codon or at the mature 3’ end of the transcript? As said, these are not the same for several transcripts. Is the section of polyadenylation representing a polyA sequence or the 3’ UTR of the RNA / the seq 3’ to the CDS? This is not explained in the legends to figures and the ref indicated does not seems to be the right one. In addition, the title of Fig 4 seems to be not the right one and in the legend to S5 Fig it is indicated “reveals effects on polyadenylation in all mRNAs” which is not the case. For example, the last column in S5 Fig present the ND6 mRNA polyadenylation. This mature transcript is about 950 nt long and the polyadenylation is at the 3’ end. Only nt 445 to 525 sequence is presented here so where is the mature 3’ end mapping system.”

We have changed the title of Fig 4 and clarified the figure legend of S5 Fig to reflect the data presented. Further clarification of these graphs is provided in response to Reviewer 2’s point 5 and we have improved the labeling of the polyadenylation plots to make the clearer based on the reviewers’ suggestion.

3) “S3E Fig. The Bru and dsRNA staining is very faint and hard to detect.”

We agree with these suggestions and have made these corrections and clarifications in the revised manuscript.

4) “What is the *ND5-CYTB* junction? Define it clearly.”

As well as all the other non-canonical junctions, the precise RNA processing site between the *ND5* and *CYTB* (the *ND5-CYTB* junction) remains unclear. However, we have suggested that an endonucleolytic cleavage occurs at the 3’-end of antisense *tRNA^E^* (Mercer *et al.*, 2011). This is consistent with the consensus that mature *CYTB* mRNA begins with a start codon at the 5′-end, and mature *ND5* contains a long 3′-UTR that elongates to antisense *tRNA^E^* (Temperley *et al.*, 2010b). Moreover, our RNA-seq data consistently suggests that the cleavage occurs around the 3′-end of antisense *tRNA^E^* (Fig 3B) (Siira et al Int J Biochem Cell Biol 2017).

5) “S4C Fig. The blot of the pre-*CO1* is too faint.”

We have provided darker versions of the blot so that the bands are more clearly visible.

6) “Ref 64 does not seem the right one for the polyadenylation method.”

Our polyadenylation mapping protocol was first used in Siira *et al.* Nature Comm 2017 and we have included information on these analyses in the methods of the revised manuscript.

**Response to reviewer #2**

1) “Non canonical is given as “mitochondria, junctions not flanked by tRNA are found at the 3’UTR of *ND6*, 5’ UTR of *CO1*, between *ND5* and *CYTB*, and between *ATP8/6* and *CO3* mRNAs. “ This is a little misleading as some of these junctions do have tRNA like structures as they are derived from the antisense of the tRNA and are predicted to fold into a very similar structure. Thus the enzyme that facilitate the cleavage may be same as for junctions between ORFs and sense strand mt-tRNAs.”

These sites are noncanonical as they do not fit with the tRNA punctuation model of mitochondrial RNA processing. Although many might have tRNA-like structures, the mechanisms of processing and the enzymes responsible have not been established for most of these sites. Even if some turn out to be processed by mitochondrial RNase P or Z enzymes they are, by definition, non-canonical as they don’t contain a true tRNA.

2) “Fig 1. Although a number of probes have been shown at 2 exposure times, a number of the signals are overexposed (eg GRSF1, VDAC) and so any more subtle differences in levels are lost. The quality of some of the probe exposures is rather poor eg FASTKD3.”

Some antibodies against abundant proteins such as VDAC1 and PNPT1 caused very strong signals even under short exposures. Therefore, we preferred not to conclude any changes in such overexposed signals, and used mtHSP70 as a reliable mitochondrial loading control.

Several commercial available antibodies (e.g. anti-FASTKD3 and anti-FASTKD5) we used were crude, and because of this technical limitation, we could not obtain any cleaner results after producing biological replicates (Fig 1B). As supplementary data, we have shown Fig I (see above), the independent biological replicate of Fig 1B.

3) “Fig 2. This is an extensive display of data but unfortunately not acceptable as essentially all of the signals are cut and paste of a single band. As a mosaic it is therefore impossible to know what was on the same blot, whether the loading was equal or represents the best from a composite of many individual blots. There are also no size markers to validate the identity of the signals. The text states that “all the analyzed antisense or ncRNAs appeared as distinct bands with precise lengths”. There are no size markers provided so it is not possible to determine is the largest species is the correct length, non-specific larger species, unprocessed, or a discrete degradation product. This is particularly important considering in most of the antisense panels there are more than one species shown. In this same section it states that “length of some ncRNAs is similar to the length of their complementary mRNAs,”. Without any size markers it is not really possible to make this conclusion. It is therefore difficult to fully assess the validity of the data presented in this figure.”

See the response to the editor’s comment 2)

The health and safety guidelines and technical limitations in the Department of Cell Biology at the University of Geneva, did not permit us to use radioactive RNA size markers for northern blotting. We have designed highly specific probes against each of the RNAs (S1 Table), and cleanly detected mRNAs and their precursor RNAs and rRNAs (Fig 2A and Fig. III below). To estimate the lengths of noncoding transcripts, each blot, where a noncoding transcript was previously probed, was subjected to re-probing against its coding counterpart, as shown in Fig. III (see below) with the example of noncoding and coding *CYTB* transcript. This way, we confirmed that all the analyzed noncoding transcripts appeared as similar lengths to their coding transcripts, thereby we defined those as full-length, processed ncRNAs. Accordingly, we named shorter ncRNAs as sncRNAs if present.

Northern blotting was used to qualitatively to determine the effects of the FASTKD deletions on rRNA, mRNA and ncRNA levels but it cannot distinguish small nucleotide changes as precisely as transcriptomic analyses or sequencing. Therefore, for some of the specific processing sites, we have applied these technologies.

Fig III. The lengths of *ncCYTB* and *CYTB* transcripts in WT HAP1 cells were compared by northern blotting. Biological triplicates were analyzed. The blot was first probed against *ncCYTB*, then against *CYTB*. The entire blot was shown.

4) “Fig 3B reflects a large amount of data. *FASTKD5* KO in Fig 3B is described as “resulted in the accumulation of transcripts or junction containing RNAs that are antisense to the *ND2*, *CO2*, *ATP8/6*, *CO3* and *ND6* mRNAs,..”. If this refers to the red in the LSP derived trace, then it is not the whole RNA species that has elevated levels in a couple of the cases and an increase (red above the line) is not really evident for ND6. If this is not what is meant then the text is not clear to this reader.”

We apologize for the oversight, we didn’t mean to include the antisense *ND6* transcript in this part of the text and we have corrected this in the revised version of the manuscript.

5) “Fig 4. The term non-template adenylation is not used in the main text that uses polyadenylation. A degree of consistency in terminology would be helpful. The change in adenylation status is not clearly described or depicted in the figure. To the less knowledgeable reader the nucleotide position might be assumed to be the nucleotide position in the mtDNA whereas it reflects the length of the mature transcript. Considering the manuscript is about non-correctly processed it would be better to relate the nucleotide position to the mtDNA position. It would also be helpful to have an indicator of the correct end following processing as this will help to align where the polyadenylation might be expected to start. The change in colour to designate this is not made clear in the legend or the text. What is also not clear is what the drop off in the graph is meant to indicate. Does it mean that *FASTKD3* KO *MTCO2* has a population of transcripts with shorter A tails ? Has this been validated by other means. If this is not what it signifies then it is not clear to this reader. This is particularly important when concluding that there was a difference in the *FASTKD4* and *FASTKD5* KOs, because this is difficult to see compared to traces for control and for the traces in the other graphs.”

We thank the reviewer for clearly articulating the required changes to clarify these graphs. We have revised this figure according to their recommendations: the numbering now shows the mtDNA position of the transcript sequence, the canonical cleavage site is labelled, followed by numbering of the non-templated poly(A) residues. The colour change indicates the region of non-templated polyadenylation and this is shown in labels on the figure and stated in the figure legend. The y axis shows the normalized read counts at each position and the drop off indicates that there was less RNA mapping to that region compared to the preceding sequence. In the case of *MT-CO2* this demonstrates that almost every transcript had at least 6 A residues added, while very few had more than 10 A residues added. The fact that the *FASTKD* knockouts had almost perfectly superimposable traces compared to WT illustrates that loss of these proteins does not impact on the abundance or polyadenylation of this mRNA. In the case of *MT-ND5* on the other hand, the normalized read counts dropped earlier for the *FASTKD* KO samples, indicating that polyadenylation of this mRNA was reduced in the absence of these proteins. This has been clarified in the revised manuscript.

6) Line 73 ‘span’ probably should be exchanged with ‘intersperse’ as span suggests coverage over the non-coding regions.

Agreed. The manuscript has been modified accordingly.

7) The lack of spacing between each transcript in Fig 3A makes it hard to take in the data. It is better presented in Fig 2.

We agree with these suggestions and have made these corrections and clarifications in the revised manuscript.

**Response to reviewer #3 (Dr. Carlos T Moraes)**

1) “I was a bit confused with the cell line “with *FASTKD3* and *FASTKD4* genes were deleted also carried a mutation in one of the *FASTKD5* alleles” HAP1 cells being haploid, the deletion of one *FASTKD5* allele would be of consequence. Maybe I am missing something.”

Please see the response to the editor’s comment 1)

2) “I was also confused with the western blots. FASTKD5 bands can still be observed in the single and double KOs. Please clarify.”

Indeed, we observed faint signals when *FASTKD5* was absent, however, they were shifted higher on the gel as shown in Fig. I above. Additionally, the commercial anti-FASTKD5 antibody we used was crude. We, therefore, concluded that these signals were non-specific to FASTKD5 protein expression. We additionally determined the modified regions by Sanger sequencing (S1 Fig) and RNA-seq (S2 Fig). The results showed that the *FASTKD5*-KO cells can express only dysfunctional truncated FASTKD5 proteins that include an N-terminal sequence of around 20 aa corresponding the mitochondrial targeting signal (27 aa in full length according to Simarro *et al.* Biochem Biophys Res Commun 2010).

3) “The observation that “degradation. The fact that the length of some ncRNAs is similar to the length of their complementary mRNAs, which suggests that the RNA processing of the two strands may occur simultaneously” is difficult to judge, as the bands do not have MW markers and are in different panels.”

Please see the response to reviewer #2 comment 3) and Figure III above.

4) “If the translatability of *ND5-CYTB* transcript is correct, should we observe a decrease in cytochrome b protein? The gels shows an overlap with ND2, but please expand. The western blot on Figure 6 does show a decrease in UQCRC2, which may be due to lower cytochrome b.”

*ND5-CYTB* precursor transcript seems to be translated, since ND5 protein synthesis was upregulated in the absence of *FASTKD4* (Fig 6A). CYTB protein synthesis may be regulated similarly, however, unfortunately, we could not observe it as the CYTB signal was overlapped with ND2 (Fig 6A). Alternatively, to measure at least stable CYTB protein expression level, we attempted to analyze the HAP1 cell lines by immunoblotting using a commercial anti-CYTB antibody. However, this experiment was unsuccessful due to the very low quality of the antibody.

5) “The binding of FASTKD4 to *tRNA^E^* is indeed interesting, but it may just reflect the fact that it binds to the unprocessed transcript at that same sequence. Still, I agree that there should be preferential binding to the unprocessed.”

The HITS-CLIP analysis allowed us to identify *tRNA^E^* as FASTKD4’s bona fide bound RNA species (Fig 5; S6 Fig), but it does not answer when this binding happens – especially before or after *tRNA^E^* is cleaved from the precursor. Future studies should address this important question as well as reveal the RNA-binding mode of FASTKD4. Extending the study to other FASTK family proteins would be also intriguing, because this RNA-binding protein family may perhaps principally bind to tRNAs to orchestrate mitochondrial RNA processing. Alternatively the FASTK family may be recruited on the LSP-derived polycistronic transcript as our previous study showed binding of FASTK protein to the *ND6* precursor transcript (Jourdain *et al.* Cell Reports 2015).
