# Supplementary material for: The FASTK family proteins fine-tune mitochondrial RNA processing
Source: PLoS Genet. 2021 Nov 8;17(11):e1009873. doi: 10.1371/journal.pgen.1009873 (PMC8601606; doi:10.1371/journal.pgen.1009873)
Supplement: Attachment — Submitted filename: Response to reviewers.docx [file pgen.1009873.s013.docx]

We would like to thank the referees for their insightful comments and suggestions on how to improve our manuscript. We think we have answered their concerns and that our manuscript has been significantly strengthened as a result.

**Response to the reviewer #2**

1) “Figure 2 is a crucial element of the manuscript containing data that is fundamental to the premise being presented. In response to comments about the fragmentary nature of Figure 2, there has been the addition of 2 sentences to state that the gel images in Fig 2 were pieces excised and from a single gel and re-assembled into a major figure. It also suggests that all the data were derived from a single gel/membrane. The example submitted to the editor/reviewers in the revision (Fig II) only shows FASTKD5-sKO suggesting that there were separate gels for each KO pairing, or where the same WT repeats used for each WT lane in Figure 2 ? The WT lanes would be expected to be identical, especially if the loading were equal and the same exposures retained, which they seem to be for the 5S. These do however differ between WT signals for many of the RNA species probed. The additional figure for the reviewers (Fig II) is not at the same exposure/brightness as the manuscript version, so it is not completely possible to judge but is it the same pair of lanes used for each probe ? Since 5S and 7SL are given as loading controls and these appear to be reasonably equally loaded, why not use WT rep3 and FASTKD5 sKO rep1. That would allow these to be presented as a pair of signals in a single piece, as was done for FASTKD3 sKO and FASTKD4 sKO data, which would be marginally better than the original and current format, instead of 2 separate pieces. WT NcND3 in fig 2 have no corresponding signal to that of the fastest mobility in the FASTKD5 sKO lane. This is different to the figure submitted to the reviewers as an example of the original gel, where the fast migrating doublet is visible in all 6 lanes. The comment about radioactive RNA markers is understood but RNA markers are available to run on the gels. Images of these on UV together with the 28S and 18S would allow for subsequent sizing to be shown.”

Every pair of signals (WT and each KO) was excised from a single gel/membrane and from the same lane and re-assembled into Fig 2A. As suggested, we have modified the pair of WT and FASTKD5-sKO in Fig 2A and shown them as adjacent lanes. Because of the overwhelming amount of samples (the large number of KO cell lines) and the technical limitations of the number of lanes we could use for a single gel run, we had to run some replicates on separate gels. For this reason, we cannot show pairs of signals from the double KO cells as adjacent lanes in Fig 2A. Instead, we provide in the revised manuscript supplementary data including two entire gels/exposures that contain all the excised signals used in Fig 2A (Fig S4).

Biological triplicates of cellular RNAs were extracted from the same cell lines. The triplicates, no matter which cell line is, would be, therefore, expected to have common molecular changes. While the loading controls, 7SL and 5S RNAs, provided comparable signals between them, signal intensities of some mitochondrial RNAs differed, particularly ncRNAs. As we previously reported, mitochondrial ncRNAs are much less abundant than their coding counterparts (Mercer et al., Cell 2011), for this reason, to make a more robust conclusion, we provided the quantification (Fig 2B-E) and used the RNA-seq as an additional approach (Fig 3).

We apologise for the WT ncND3 oversight in Fig 2A, this has been corrected for the pair of WT and FASTKD5-sKO in Fig 2A and in Fig II (WT rep 3 and FASTKD5-sKO rep1). All data shown in Fig 2 and S4 Fig, show samples from the same gel for each probe.

2) “Fig 4 seems to suggest that the poly(A) tail in WT controls is generally 15 nucleotides, which does not correspond with most of the literature including that cited by the authors in their response (Temperley et al 2010).”

The polyA analyses were done to investigate the effects of FASTKD protein deletions on the processing of mRNAs and specifically investigate if that would affect their oligoadenylation. These analyses are not challenging the currently identified lengths of mt-RNA polyA tails, as shown previously in Temperly et al., they are merely a readout of any potential defects at the 3’ end of mitochondrial transcripts and if this affects their adenylation status. The limitation of high throughout sequencing analyses for polyadenylation precludes from confidently defining each of the ends. Specifically, next generation sequencing analyses are limited by the inherently short read lengths produced and as 3’ end mapping requires significant matching to the 3’ mRNA body as well as the subsequent oligoadenylated regions, the full lengths of the polyadenylated sequences are not able to be quantitated exactly.
